# Simultaneous Quantification of Steroid Hormones Using hrLC-MS in Endocrine Tissues of Male Rats and Human Samples

**DOI:** 10.3390/metabo12080714

**Published:** 2022-07-30

**Authors:** Guillermo Bordanaba-Florit, Sebastiaan van Liempd, Diana Cabrera, Félix Royo, Juan Manuel Falcón-Pérez

**Affiliations:** 1 Exosomes Laboratory, Center for Cooperative Research in Biosciences (CIC bioGUNE), Basque Research and Technology Alliance (BRTA), 48160 Derio, Spain; froyo@cicbiogune.es; 2 Metabolomics Platform, Center for Cooperative Research in Biosciences (CIC bioGUNE), Basque Research and Technology Alliance (BRTA), 48160 Derio, Spain; smvanliempd@cicbiogune.es (S.v.L.); dcabrera@cicbiogune.es (D.C.); 3 Centro de Investigación Biomédica en Red de Enfermedades Hepáticas y Digestivas (Ciberehd), 28029 Madrid, Spain; 4 Ikerbasque, Basque Foundation for Science, 48011 Bilbao, Spain

**Keywords:** liquid chromatography–mass spectrometry, time-of-flight, steroid hormones, androgens, urinary extracellular vesicles, hormone-dependent disease, metabolomics

## Abstract

Steroid hormones play a vital role in the regulation of cellular processes, and dysregulation of these metabolites can provoke or aggravate pathological issues, such as autoimmune diseases and cancer. Regulation of steroid hormones involves different organs and biological compartments. Therefore, it is important to accurately determine their levels in tissues and biofluids to monitor changes after challenge or during disease. In this work, we have developed and optimized the extraction and quantification of 11 key members of the different steroid classes, including androgens, estrogens, progestogens and corticoids. The assay consists of a liquid/liquid extraction step and subsequent quantification by high-resolution liquid chromatography coupled time-of-flight mass spectrometry. The recoveries range between 74.2 to 126.9% and 54.9 to 110.7%, using a cell culture or urine as matrix, respectively. In general, the signal intensity loss due to matrix effect is no more than 30%. The method has been tested in relevant steroidogenic tissues in rat models and it has also been tested in human urine samples. Overall, this assay measures 11 analytes simultaneously in 6 min runtime and it has been applied in adrenal gland, testis, prostate, brain and serum from rats, and urine and extracellular vesicles from humans.

## 1. Introduction

**Figure 1:**
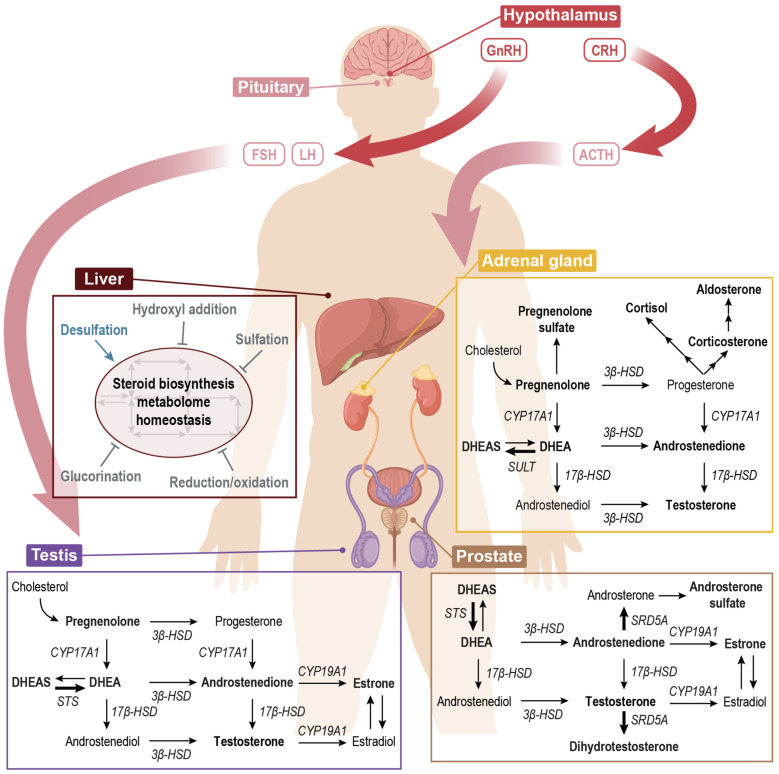
Schematic representation of the steroid hormone biosynthesis pathway in relevant organs and its regulation. CRH stimulates the release of ACTH from the pituitary gland. ACTH stimulates the production of cortisol (exerts negative feedback on CRH and ACTH) and DHEAS in adrenal glands. Pulses of GnRH from hypothalamic neurons stimulate pulses of LH as well as FSH. LH stimulates testosterone production in testis. Liver maintains pathway’s homeostasis and several processes may happen: sulf desulfation makes metabolites available to feed the pathway while processes indicated with a flat end arrow inactivate metabolites that are in circulation. Bold arrows indicate a higher activity of the specific reaction. In bold, the metabolites that are majorly produced in each specific organ are represented. ACTH: adrenocorticotropin; CRH: corticotropin-releasing hormone; FSH: follicle stimulating hormone; GnRH: gonadotropin-releasing hormone; LH: luteinizing hormone; CYP17A1: Steroid 17-alpha-monooxygenase; CYP19A1: aromatase; SULT: hydroxysteroid sulfotransferase; STS: steroid sulfatase; 3β-HSD: 3β-Hydroxysteroid dehydrogenase; 17β-HSD: 17β-Hydroxysteroid dehydrogenase; DHEA: dehydroepiandrosterone; DHEAS: DHEA sulfate.

Steroid hormones are involved in a wide range of physiological processes and their production and delivery is regulated via the hypothalamus–pituitary–adrenal gland and –gonadal axes (Figure 1) [1]. Regulation is, amongst other things, subject to circadian rhythm, stress and sex. There are five classes of steroid hormones, namely glucocorticoids, mineralocorticoids, progestogens, androgens and estrogens. These different classes have distinct biological functions. The glucocorticoids are involved in the stress and immune response, while the mineralocorticoids are more related the maintenance of cell homeostasis [1,2]. In addition, the androgens and estrogens highly regulate cellular proliferation, development and differentiation. Hence, dysregulation of the steroid signal cascades often results in hormone-dependent pathologies. For instance, the carcinogenesis of breast and prostate cancer (PCa) are strongly influenced by the systemic presence of active estrogens [3] and androgens [4,5], respectively. Specifically, in PCa, the androgen receptor triggers the tumorigenic growth at a molecular level. The active steroid hormones, such as 5-α dihydrotestosterone (DHT), are the major ligands in this molecular pathway and cause the progression of PCa at early stages [4,6].

In mammals, the precursor of sterol biosynthesis is cholesterol, which is further utilized in the adrenal glands, gonads and sexual-derived tissues to produce steroid hormones. There are 99 metabolites involved in the steroid hormone biosynthesis pathway and over 100 reactions are catalyzed by 61 different enzymes [7,8]. All of the steroid compounds share a sterane backbone structure. The physiological role of each individual steroid hormone is primarily defined by the layout of double bonds, hydroxyl and keto groups around this basic sterane backbone structure [1]. The main structural difference between the classes is the carbon atom arrangement i.e., the androgens are C-19, the estrogens are C-18, the progestogens are C-20 and the corticoids are C-21.

In the first step of the steroid hormone biosynthesis, cholesterol is internalized into the mitochondria where it is fed as a substrate to produce pregnenolone (Figure S1, Supplementary Materials). This is the main precursor for steroid hormones produced de novo [4] inside the mitochondria. Pregnenolone can be converted to progesterone or dehydroepiandrosterone (DHEA), which can be further metabolized to glucocorticoids and mineralocorticoids (C-21) or to androgens (C-19), such as testosterone, DHT or androsterone and estrogens (C-18), respectively (Figure S1, Supplementary Materials). Interestingly, this metabolic network is tissue-dependent. Different organs are specialized on particular modules of the pathway that are physiologically relevant to perform their function. For instance, the adrenal glands are the producers of C-21 hormones, while prostate shows a high SRD5A activity, which catalyzes the conversion of testosterone to DHT (Figure 1).

Indeed, this is an intricate network of metabolites. Many of these metabolites participate as ligands in a wide span of signaling cascades and biological processes, and their levels vary strongly between different biological compartments. While cholesterol is the unique de novo precursor in steroid hormone biosynthesis, there exists an interchange between cells and tissues that anaplerotically feeds the pathway at the intermediate steps [9]. This means that the compounds upstream of the pathway can be provided by the cell environment. In this line, sulfated steroids are of interest since they are, unlike their unsulfated counterparts, readily soluble in the cytoplasm and in biofluids, such as blood or urine. Notably, the sulfates of steroids are considered endogenous and active neurosteroids [9,10]. Over the past few decades, it has been established that sulfonation is not only a process to inactivate and excrete steroid hormones; it also acts as a systemic reservoir for peripheral or local steroidogenesis in non-steroidogenic tissues, i.e., the brain or prostate [9,11]. In addition, it has been reported that the secreted vesicles, also known as extracellular vesicles (EVs), participate in many of the physiological processes [12,13] and they can contain a wide variety of cargos, such as lipids, proteins, metabolites, sugars and even DNA [12,13,14,15]. The hormone steroids and related cargos are transported by the blood and other body fluids as sulfated species, but they could also be transported by EVs to reach the target tissues.

The steroid hormone metabolism and the consequences of dysregulation have gained interest within the biomedical community to understand and diagnose hormone-dependent diseases, rather than the historic usage of steroid hormones in therapeutics. Indeed, a number of methods to detect and quantify steroid hormones have been reported during the last two decades. Many of the studies describe methodologies to detect steroids from several biological sources: cell cultures [3,16,17]; urine samples [18,19,20]; animal tissues [21,22,23]; human serum [24,25,26]; human hair [27] and waste water [28,29]. In general, steroid metabolomics methodologies focus on profiling a specific set of metabolites of interest in targeted tissues (or in circulation) rather than analyzing steroidogenesis status in a system of organs and related fluids. The methods are usually developed for similar non-sulfated steroids that efficiently ionize in the same mode, avoiding the exploration of the detection and quantification of many different steroids simultaneously [16,23,25,26]. Methodologically, these studies describe a variety of extraction, separation and detection methods. In particular, the solid phase extraction (SPE) and reversed phase liquid chromatographic-based methods are deployed in the isolation and separation of these compounds. The detection is mostly performed with triple quadrupole instruments. In addition, gas chromatography-coupled MS methods was also utilized in a few of the studies. All of these methods have their advantages and disadvantages. 

We describe a method for the detection of endogenous steroid hormones and their intermediates, using liquid/liquid extraction and ultra-performance liquid chromatography (UPLC), coupled with high resolution time-of-flight mass spectrometry (hrLCMS). UPLC provides fast cycling times and a high chromatographic resolution. The high mass resolution obtained with time-of-flight mass spectrometry results in high specificity, while the sensitivities are on par with triple quadrupole methods. This method was applied to metabolically profile several animal tissues and urinary EVs (uEVs). Different biological matrices, including prostate, adrenal gland, testicles, brain and liver of Wistar male rats but also human urinary samples, were tested in this assay. To our knowledge, the present work presents for the first time a reliable and optimized hrLCMS assay to analyze the key endogenous steroid hormones in endocrine tissue, bioliquids and EVs.

## 2. Materials and Methods

### 2.1. Tissue and Biofluid Samples

The tissues and serum were obtained from three wild-type (Wistar, RjHan:WI) rats obtained from Janvier Labs, Le Genest-Saint-Isle, France. All of the urine samples were obtained from a healthy male on either the morning or the afternoon. uEVs were obtained by ultracentrifuging urine samples as described elsewhere [5]. Urine samples and uEVs were characterized in several physicochemical parameters and protein markers, respectively. For a more detailed information on sample collection, preparation and characterization refer to Figure S1 (Supplementary Materials). 

### 2.2. Chemicals and Standards

The DHEA, DHT, cortisol (in methanol solution) and the sodium salt of androsterone sulfate were obtained from Cerilliant Corporation (Round Rock, TX, USA). Supelco (Bellefonte, PA, USA) procured androstenedione. The sodium salts of DHEAS and pregnenolone were obtained from Avanti Polar Lipids, Inc. (Alabaster, AL, USA). The testosterone, aldosterone, corticosterone, estrone, pregnenolone 3-sulfate (sodium salt form), leucine-enkephalin (Leu-Enk), chloroform (>99.8% pure; of chromatography grade) and ammonia solution were purchased from Sigma-Aldrich (St. Louis, MO, USA). The LC-MS grade water, acetonitrile, formic acid and methanol were purchased from Fisher Chemical (Fair Lawn, NJ, USA). 

### 2.3. LCMS Sample Preparation

The steroid metabolites were extracted by liquid–liquid extraction using a methanol/water mixture and chloroform as extraction liquids. The EV fractions were sonicated for 15 min in a total volume of 400 µL 50% *v*/*v* methanol/water mixture containing 1 mM ammonia to lysate EVs. The cell culture (DU145 cell line), fixed on culture well plates, was scrapped after 5 min incubation with 500 μL 50% *v*/*v* methanol/water mixture containing 1 mM ammonia. Tissue aliquots—approximately 50 mg—were lysed, using 1.4 mm zirconium oxide beads into standard 2 mL homogenizer tubes (Precellys, Montigny, France). Each sample was homogenized in 500 μL 50% *v*/*v* methanol/water mixture containing 1 mM ammonia by performing two cycles of 40 s at 6000 rpm in a FastPrep-24TM 5G bead beating grinder (MP Biomedicals, Solon, OH, USA). After lysis, 400 μL of the homogenate—either tissue, EV fraction or DU145 cell culture—was transferred to a clean Eppendorf^®^ tube. Subsequently, 400 μL of LCMS grade chloroform was added on top of the 400 μL of any lysated sample and shaken for 60 min at 1400 rpm at 4 °C. Then, the samples were centrifuged for 30 min at 14,000 rpm at 4 °C in order to precipitate the proteins and to separate the organic from the aqueous phases. 

The aqueous (top) and organic (bottom) phases were separated. The protein fraction was precipitated on the meniscus between these two immiscible phases. Then, 250 μL of each fraction was transferred to the clean Eppendorf^®^ tubes and evaporated using a centrifugal vacuum concentrator. The pellets from the organic fraction were dissolved in 100 μL pure methanol and the pellets from the aqueous fractions were dissolved in 50% *v*/*v* methanol/water. All of the resuspended pellets were centrifuged for 30 min at 13,000 rpm and 4 °C. Finally, 80 μL of the resuspended pellets were transferred to deactivated glass vials or 96-well plates for injection into the hrLCMS system.

### 2.4. Ultra-High Performance Liquid Chromatography (UPLC)

The chromatographic separation of the analytes was performed with an ACQUITY UPLC I-Class PLUS System (Waters Inc., Milford, MA, USA). This system was equipped with a cooled (10 °C) Process Sample Manager with a sample loop of 10 µL and a Sample Organizer, a Binary Solvent Manager and a High Temperature Column Heater. A reversed-phased 1.0 mm × 100 mm BEH C18 column (Waters Inc., Milford, MA, USA), thermostated at 40 °C, was used for separating the analytes. The samples were injected from either 2 mL deactivated glass vials or 700 µL round 96-well polypropylene plates. 

The chromatographic behavior was optimized with respect to the peak intensity and an adequate separation of the 11 analytes along the run. The gradient elution was accomplished with an aqueous mobile phase (eluent A) consisting of 99.9% water with 0.1% formic acid and an organic mobile phase (eluent B) consisting of 99.9% acetonitrile with 0.1% formic acid. The flow rate was 140 µL per min. Several gradients were tested during the optimization process (Table S1, Supplementary Materials) in order to avoid break-through (elution of analyte in the injection peak) and to obtain a good peak separation. The optimal gradient was as follows: start at 30% B; a linear increase to 80% B in 3.8 min.; a step increase from 80% to 99%; constant at 99% for 1.0 min and back to 30% B in 0.2 min. The total cycle time from injection to injection was 6 min. The injection volume for all of the samples was 2 µL. 

### 2.5. Mass Spectrometry

A time-of-flight mass spectrometer SYNAPT G2-S (Waters Inc.) was utilized for the detection of the analytes. The instrument was operated in either positive (ESI+) or negative (ESI-) electrospray ionization mode and in full-scan mode with a scan range between 50 Da and 1200 Da and scan time of 0.2 s. 

The z-spray source parameters: temperatures; gas flows; capillary position and voltages were tuned, as detailed elsewhere [30]. The optimal source parameters for this assay in either ESI+ or ESI− are summarized in Table S2 (Supplementary Materials). The ion optics were fine-tuned by spraying Leu-Enk (100 ppb), at a rate of 10 µL per min, to a resolution over 20,000 (FWHM) for *m*/*z* 556.2771. The same Leu-Enk solution was sprayed as a lock mass to correct for *m*/*z* fluctuations along the assay. The lock mass solution was introduced into the source every 90 s using a second ESI probe and it was recorded for 0.5 s. Mass spectrometer spectra was corrected according to fluctuations detected in the lock mass. 

### 2.6. Statistical Analysis

#### 2.6.1. Analyte Recovery Study

The extraction step efficiency was assessed by performing a recovery assay with various mixtures of organic solvents and water. Five different extraction buffers were tested in this assay: 25/75% *v*/*v* and 50/50% *v/v* of methanol/water mixture; 25/74.9/0.1% *v/v/v* and 50/49.9/0.1% *v/v*/*v* of methanol/water/formic acid mixture and 50/50% *v/v* of methanol/water mixture with 1mM ammonia. To compare and calculate the recoveries of 10 different analytes, a culture of a prostate cancer cell line-DU145-was spiked with the analyte standards. Each well containing 5·× 10^5^ cells was spiked with a mix of standards at 2 µM before lysis (pre-spiked) and at the resuspension stage (post-spiked) with a standard mix at 10 µM. Thus, the pre-spikes contained 1 nmol in 500 µL and post-spikes (aqueous and organic fractions) contained the same total amount in 100 µL, which would be the theoretical maximum absolute if there was no loss during the extraction. In addition, for each extraction solution, the non-spiked samples were prepared in order to correct for endogenous metabolites in the matrix. The samples for the pre-spiked, post-spiked and non-spiked conditions and the five different extraction buffers were prepared in biological triplicates. 

Only the absolute peak areas were taken into consideration to establish the recovery efficiency in the extraction step. The average peak areas were obtained by mean smoothing the raw signals of triplicates. The recovery (*R*) was determined by dividing the corrected pre-spike average by the corrected post-spike average and represented as a percentage (Equation (1)). Both the pre-spiked and post-spiked raw signals ought to be corrected by subtracting the endogenous analytes signal in the DU145 culture matrix (*S_non-spike_*). However, as the *S_non-spike_* of DU145 culture matrix was less than 0.05% of the signal, endogenous correction was neglected during the calculation. Importantly, the pre-spikes were corrected with respect to analyte loss (*α*) during the extraction procedure. Moreover, the raw signals of each sample did not have to be corrected by the amount of initial samples, because every well contained the same amount of cells.

(1) $$R\;\left(\%\right)=\frac{\alpha \left(S_{pre-spike}-S_{non-spike}\right)}{S_{post-spike}-S_{non-spike}}\times 100$$

#### 2.6.2. Study of Matrix Effect in Analyte Quantification

In order to assess the matrix effect (*ME*) in the quantification of the analytes, the post-spiked raw signal was compared to an equivalent raw signal of a mixture of analytes (10 µM) in solution. The post-spiked raw signals were corrected by subtracting the endogenous analytes detected in the non-spiked DU145 culture samples. Then, the numerator was divided by the average peak areas of the standards and expressed as a percentage (Equation (2)):

(2) $$ME\;\left(\%\right)=\frac{S_{post-spike}-S_{non-spike}}{S_{standards}}\times 100\;$$

#### 2.6.3. Analyte Semi-Quantification

In this work, a calibration curve was prepared in solution with 50% *v/v* methanol/water for the semi-quantification of the analytes. This calibration curve consisted of a serially diluted mixture containing all of the analytes, starting at a concentration of 10 µM. The initial concentration was diluted to half concentration twice, resulting in 5 µM and 2.5 µM concentration in the curve. Then, this set of triplets was diluted in five decades; it resulted in the following 15 different concentrations per analyte: 10; 5; 2.5; 1; 0.5; 0.25; 0.1; 0.05; 0.025; 0.01; 0.005; 0.0025; 0.001; 0.0005 and 0.00025 µM. The calibration samples were injected at the beginning and at the end of each experiment; the average of these two points was used to semi-quantify the metabolites in the tissues.

The limit of detection (LOD) for each analyte was set to be the lowest concentration at which the signal-to-noise (S/N) ratio was above three. The LOQ was defined as the lowest concentration at which the S/N ratio was above 10. The highest quantifiable concentration was the highest concentration per analyte that fits the calibration curve with an acceptable accuracy and precision (CV ≤ 15%) [16].

In general, the data of a calibration curve range over several orders of magnitude, the data are not linear and tend to be heteroscedastic [31]. For this reason, the relation between the peak area and the sample concentration was determined by power-fitting [30]. The power fitting resulted in a calibration curve (Equation (3)) with *α* and *b* as the fitted parameters. Once the sample concentrations were calculated using a calibration method in solution, the amount (in nanomole) per gram of tissue weight was estimated:

(3) $$Peak\;area=\alpha {\left[concentration\right]}^{b}$$

## 3. Results

### 3.1. Liquid Chromatography and Mass Spectrometry Method

We compared six different chromatographic methodologies (Table S1, Supplementary Materials) to satisfactorily separate the analytes. The gradient 6 (30% B to 80% B in 3.8 min; detailed steps in Table S2, Supplementary Materials) showed the best peak separation along this run time compared to other tested gradients (data available in [32]). Due to the nature of the stationary phase, analytes elute in order of increasing hydrophobicity. The resulting extracted ion current (XIC) chromatograms of a standard mixture at 10 µM are depicted in Figure S2 (Supplementary Materials). In brief, aldosterone (*m*/*z* 361.2015; ESI+) elutes at 0.99 min, cortisol (*m*/*z* 363.2171; ESI+) at 1.20 min, DHEAS (*m*/*z* 367.1579; ESI−) at 1.60 min, corticosterone (*m*/*z* 347.2222; ESI+) at 1.68 min, androsterone sulfate (*m*/*z* 369.1736; ESI−) at 1.85 min, pregnenolone sulfate (*m*/*z* 395.1892; ESI−) at 2.23 min, estrone (*m*/*z* 271.1698; ESI+) at 2.39 min, androstenedione (*m*/*z* 287.2011; ESI+) and DHEA (*m*/*z* 289.2168; ESI+) co-elute at 2.40 min, DHT (*m*/*z* 291.2324; ESI+) at 2.65 min, pregnenolone (*m*/*z* 317.2481; ESI+) at 3.25 min. 

Regarding the mass spectrometry method, the Leu-Enk signal (*m*/*z* 556.2771) was aimed at a resolution of over 20,000 (FWHM) and provided the necessary mass accuracy to evaluate assay analytes. Isotope pattern matching and the use of chemical standards confirming elution times further ensured the specificity. In general, the mass accuracies for the analytes in solution were between −1 to 1 mDa. It is noteworthy that several analytes were not adequately separated during the chromatographic elution. The corticosterone and DHEAS elute at similar retention times—1.60 min and 1.68 min-, however, the MS could properly distinguish them by their *m*/*z* difference and their fragmentation pattern. Moreover, the DHEAS was not detected with a high intensity signal in ESI+ mode. For this reason, the corticosterone was measured in ESI+ and the DHEAS in ESI− mode. Likewise, estrone, DHEA and androstenedione eluted in approximately 2.40 min. In this case, one could only rely on the MS sensitivity (estrone *m*/*z* 271.1698, DHEA *m*/*z* 289.2168, androstenedione *m*/*z* 287.2011) and on a fragmentation pattern that was sensitive enough to distinguish and quantify them separately.

### 3.2. Analyte Recovery Optimization

Afterwards, we evaluated the recovery of 11 analytes using a biphasic liquid–liquid method and analyzed them with the optimized hrLCMS method. The extraction was performed, using the DU145 cell line as a matrix. Five different mixtures of organic solvents and water, containing either formic acid or ammonia to modify the pH of the extraction buffer or no pH modifier, were assessed (Table S3, Supplementary Materials). The addition of formic acid strived for lowering the pH approximately to three, while 1mM ammonia modified the extraction buffer to pH 8–9 in order to chemically neutralize the functional groups of the steroid compounds. From the previous experiments in our metabolomics platform, we observed that in liquid–liquid extraction requires at least 25% organic solvent during the extraction step to precipitate the proteins. This is important to avoid clogging the chromatographic system [30]. Moreover, the effectivity of tissue homogenization using beads has been reported as high and does not differ much from the homogenization of other matrices, such as urine or cell cultures [30,33]. Therefore, the calculated recoveries are ultimately dependent on the extraction buffer utilized, regardless of the homogenization methodology. 

During the optimization process, it was determined that the steroid sulfate compounds were recovered completely in the aqueous fraction, whilst steroids without sulfate group were found in the organic fraction. Notably, only cortisol was detected systematically in both of the fractions (Figure S3, Supplementary Materials); however, it was majorly recovered in the organic (80% or higher) rather than in the aqueous (approximately 20%) fraction. Moreover, the addition of formic acid to the extraction buffer led to a dramatic decrease in the recoveries of the sulfate compounds and a slight decrease in the rest of the steroid analytes (Figure S3, Supplementary Materials). One can infer that the presence of protons in the buffer do not stabilize steroid charges and severely hampers the extraction of sulfate steroids in a polar environment. The supplementation of 1mM ammonia outperformed the extraction in terms of recovery and robustness, compared to the other extraction liquids. Notably, the recovery values using different percentages of methanol in the extraction buffer do not differ much. However, the extraction efficiency of the sulfate compounds using 25% *v/v* methanol underperforms 50% *v/v* methanol, with a recovery loss of 40 to 50%.

In Table 1, the recoveries of the 11 selected analytes, using a mixture of 50/50% *v/v* methanol/water with 1mM ammonia as the extraction buffer, are reported. In general, the present methodology is able to recover and detect over 90% of the initially spiked analyte. Only DHT was detected in a lower percentage; approximately 80% of the initially spiked DHT was recovered. As expected in a biphasic extraction, the hormone steroids were retrieved in an apolar environment and the sulfated steroids in a polar solvent. Besides cortisol, pregnenolone sulfate was also reported in both of the fractions; it was mainly recovered in the more polar solvent and a derisory amount in the organic fraction. Using this methodology, the recoveries for 10 µM of analyte ranged from 74.2% to 126.9%. These values are acceptable for routine muti-analyte hrLCMS analysis since all of the results are reproducible [34]. Thus, extraction using 50/50% *v/v* of methanol/water mixture with 1 mM ammonia was selected for further experiments in different biological matrices.

Furthermore, the performance of the optimized methodology was tested, using urine as the matrix since it has a high interest for clinical applications. Six samples of urine from a male individual were pooled and aliquoted in different two volumes to assess the matrix effect on the recovery efficiency. In Table 2, the recoveries of the 10 analytes are reported; DHEA recovery has not been retrieved, because its peak was masked by testosterone’s signal. In general, over 85% of the initially spiked analyte is recovered and detected in 50 µL urine matrix. Importantly, the sulfated steroids are not recovered with the same efficiency; DHEAS and pregnenolone sulfate report a recovery efficiency of 75.7% and 54.9%, respectively. The recoveries of the analytes using 250 µL urine as matrix describes a slight decrease in the non-sulfated steroids while the efficiency decay is dramatic in the sulfated species. 

### 3.3. Matrix Effect

It is well known that the phospholipids and other lipids, typically enriched in biological matrices, such as tissues, body fluids or cell cultures, can cause ion suppression in mass spectrometry, thereby hampering the analyte signal [35,36]. This phenomenon negatively influences the detection of the analytes and may underestimate their quantification. For a specific matrix, the higher the ion suppression effect is, the higher the signal loss. Therefore, the conclusions drawn by detecting and quantifying the analytes under these conditions could be misleading.

The matrix effect of each analyte was defined as the signal loss measured at the resuspension step (sample spiked with 10 µM analyte mix) compared to 10 µM of each analyte in solution. The signal loss was calculated in five different extraction procedures, because they can influence ion suppression. The matrix effect reported in this work was estimated for a prostate cancer cell line (DU145) culture and urine samples. To note, signal loss is specific for each matrix and each independent experiment. In further experiments, in which quantification is required, the matrix effect should be calculated in every particular assay. From our optimization experiments, one can infer that the matrix effect is fraction-dependent, because there is a significant difference between signal loss comparing organic and aqueous fractions (Figure S4, Supplementary Materials). This phenomenon is likely observed due to a differential extraction of the phosphatidylcholine (or other lipid) compounds [30,35]. Strikingly, this fraction dependency was not observed upon the addition of ammonia to the extraction liquid. Moreover, the presence of ammonia resulted in a signal loss of up to half-fold compared to extraction liquids with acidic modifier or no pH modifier addition. This suggests that the ammonia impairs the extraction of the lipidic compounds from the biological matrix, hence, decreasing the ion suppression phenomenon in mass spectrometry. 

In Table 1, the matrix effect (expressed as signal loss (%)) of a DU145 culture of 11 selected analytes, using a 50/50% *v*/*v* of methanol/water mixture with 1mM ammonia for extraction, is reported. In general, the present methodology loses approximately 15 to 40% of the signal of non-sulfated analytes but it mainly lays between 20 to 30% loss. On the other hand, the sulfated steroids display a 40 to 50% loss of signal, regardless of the extraction fraction. The signal loss of the 10 µM analytes spiked in DU145 cell line were: 25.2% for pregnenolone, 37.7% for DHEA, 30.8% for androstenedione, 25.5% for estrone, 23.1% for DHT, 25.9% and 20.2 % for cortisol in the organic and aqueous fraction, respectively, 18.6% for aldosterone, 25.0% for corticosterone, 46.1% for pregnenolone sulfate and 42.5% for DHEAS. All of the analytes are majorly recovered back in a particular fraction of the extraction procedure, which is the one selected to report the matrix effect. Signal loss of sulfate compounds refer to aqueous fraction measurement and the other steroids refer to signal loss in organic fraction. 

### 3.4. Semi-Quantitation of Steroids in Animal Tissues

The hrLCMS method was most sensitive in detecting androstenedione, DHT, corticosterone, pregnenolone sulfate and DHEAS with a LOD (S/N > 3) of 250 pM in a 50/50% *v*/*v* methanol/water solution. The detection limit for cortisol and aldosterone was 0.5 nM, and a LOD of 2.5 nM was determined for pregnenolone. The least responsive ions were those for DHEA and estrone with a LOD of 5.0 nM. With regards to the quantification limits, androstenedione and DHEAS were the most sensitive compounds, with a LOQ (S/N > 10) of 0.5 nM in solution. The cortisol, corticosterone, pregnenolone sulfate and DHT were in the second group of the most quantifiable ions showing a LOQ of 1.0 nM. The quantitation limit for aldosterone was 2.5 nM, while a LOQ of 0.01 µM was estimated for pregnenolone and estrone. The DHEA was the compound with the highest quantitation threshold (0.05 µM). 

We found that the concentration range of the steroid hormones is typically low in tissues, ranging from pico- to nanomole per gram of tissue, and cannot be detected in some tissues (Table 3). Only pregnenolone, androstenedione, DHT, corticosterone, cortisol and testosterone were detected in the tissues or serum of Wistar rats. Pregnenolone and cortisol are only quantified in the adrenal gland tissue, however, pregnenolone is also detected in the brain and testicles. Adrenal gland and testicles reported picomole amounts of androstenedione per gram of tissue. Moreover, DHT was quantified in the prostate, adrenal gland and testicles. In prostate, the amount of DHT was two-fold the quantitation in the other tissues. The testosterone and corticosterone were quantified in all of the measured rat samples. In general, they were reported in the picomole per gram range in tissues. In serum, they were quantified in the nM range. Interestingly, the adrenal gland described nanomole per gram concentrations of corticosterone. Furthermore, testosterone was found in a one order of magnitude higher amount in the adrenal gland and testicles compared to prostate and brain. 

The standard deviations and coefficients of the variation are rather large, indicating an important variability among the samples obtained from the same strain but independent animals. One could expect this biological variation and it suggests that treatments, stress or any procedure applied to animals can potentially influence the outcome in further experiments. 

### 3.5. Quantitation of Steroid Hormones in Human Urinary Samples

Six different urine samples were characterized in several physicochemical parameters (Table S4, Supplementary Materials) to examine whether the sample collection resulted in homogenous sample groups, regardless of the metabolomics’ analysis. No blood, ketone bodies or glucose were detected in the urine sample, and the pH value and density of the urine were similar in all of the samples. The urine samples were centrifuged in two serial steps at 10,000× *g* for 30 min to isolate the so-called P10K fraction—typically containing vesicles of 150 to 200 nm diameter and above—followed by a 100,000× *g* centrifugation for 90 min to isolate the so-called P100K—typically containing vesicles of 100 to 150 nm diameter and below (up to 50 nm) [37]. The supernatant of the second centrifugation was also analyzed and referred to as SN100K. 

In this set of urine samples, the current methodology is able to detect and quantify androstenedione, cortisol and DHEAS (Table 4). The other steroids of the panel were below the LOQ and, in general, also below the LOD. The androstenedione and cortisol were detected only in the urine and SN100K. It was not possible to detect them associated with the EVs, and they are majorly solubilized in the urine. The androstenedione was found in lower concentrations compared to cortisol and the variability between the collection days was high (40 to 60%) regardless of the collection time. Concerning cortisol, the variability was extremely high between the morning collection days (approximately 50 to 85%) whilst the concentration of the afternoon collected samples was stable (approximately 2% variation). DHEAS was the compound detected in the highest concentration (µM range) soluble in urine, compared to androstenedione and cortisol (nM range). Similar to androstenedione, the DHEAS showed a high variability over independent collection days at both the morning and afternoon collection times. To note, DHEAS was the only metabolite detectable in the EV fraction. In Table 4, the absolute amount (µmol) in 50 mL of urine is reported but also the relative amount (in ppm) of the total detected metabolite that is associated with the EVs. Importantly, DHEAS was not quantifiable (S/N < 10) in all of the samples collected at morning time, but it was detectable in all of the cases (S/N > 3). According to our analysis, a range of 0.5 to 3.0 ppm of DHEAS was associated with the EVs in the urine samples (Table 4; detailed calculations available in [32]). 

The isolation of the EVs in the pellet fractions was confirmed with the presence of typical EV markers by Western blotting (Figure S5, Supplementary Materials). Typical urine exosome markers, such as CD9, CD63 and AQP2, were intensified in P100K fractions, confirming that this fraction is enriched in EVs. However, they are sample-dependent and were detected in various amounts. In addition, LAMP2A and CD10 were detected only in the P100K fraction of U003-derived EVs preparation. Annexin V and AQP2 were found in both P100K and P10K, but also in different amounts among urine samples. 

## 4. Discussion

This work describes a fast and simple hrLCMS methodology, able to detect and quantify 11 key metabolites of the steroid hormones biosynthesis in several biological matrices. Their importance in diseases, such as PCa and other steroid-dependent diseases, spotlights this assay as a powerful tool to study the role of steroid hormones in the development and progression of hormone-dependent diseases and to assess the metabolic status of patients via liquid biopsy analysis. In brief, this method identifies and quantifies 11 steroids, including corticoids, androgens and metabolic intermediates, in a high-throughput method of 6 min. Although testosterone and androsterone sulfate were not included in the recovery experiment, the methodology is able to separate, identify and quantify them. 

All of the steroid hormones are primarily derived from cholesterol, which provides the sterane ring structure shared by all of these compounds (Figure S1, Supplementary Materials). Subtle chemical differences, unique to each steroid hormone, significantly complicate the separation of such structurally similar molecules. Furthermore, the structure of the steroids and position of the functional groups determine their preferred ionization mode and efficiency [18,24]. For instance, testosterone and DHEA—with the same molecular formula—display different ionization efficiencies. DHT or androstenedione are readily ionized in positive mode, in contrast to DHEA or pregnenolone, which are not strongly ionized due to the presence of keto groups in the ionizable region (Figure S1, Supplementary Materials). In order to increase the signal intensity, the MS could be operated in enhanced duty-cycle (EDC) mode; this is a more appropriate approach in targeted analyses, where the analyte empirical formulas are known. In this strategy, the MS signals of a given retention time are measured in separate scan functions to enhance the *m/z* of the selected analyte. Measuring in EDC instead of full-scan mode may increase by several fold the S/N ratio of a given metabolite [30,38,39]. Therefore, the EDC mode is an option to consider for those samples in which the analytes S/N ratio falls above the LOD, but are not always quantifiable. 

An LCMS method is usually evaluated in terms of efficiency, accuracy and sensitivity of the measurement. The process efficiency is a combination of recovery efficiency and matrix effect of each metabolite [40], and the sensitivity is evaluated with the LOD and LOQ of each metabolite. Different studies identifying and quantifying steroid compounds in biological matrices report a wide range of efficiency recoveries. For example, in PCa cell cultures, a recovery range of 54.7% to 78.1% was reported [16] while in breast cancer cell cultures, recoveries ranging 95.7 to 102.0% were reported [16]. Our data, with recoveries ranging from approximately 75% to 125%, suggest that a cell culture as matrix does not impair the extraction of the steroid metabolites. The urine matrix does not impair the extraction of the non-sulfated steroids but the sulfated species suffer a recovery efficiency decay. To note, the studies measuring steroids in urine and tissues, as biological matrices report recovery efficiencies of over 100% in some of the cases [17,18,19]. An explanation for this phenomenon might be that the metabolites can be either free in solution or tethered to other molecules, such as membranal lipids during the extraction process. For this reason, the organic and aqueous phase recoveries are not adding up to 100% in this assay. In case of detecting a metabolite in two fractions, the addition of both of the signals is perhaps a better approach to quantify that specific metabolite. However, our assay is very convenient, since all of the metabolites (except cortisol) are recovered in only one fraction. This permits a faster measurement of the steroid hormones in different biological matrices.

The existing quantitation methods for steroid hormone compounds have a wide span of LOQ, ranging from 0.002 to 10 ng per mL. However, it is highly dependent on the analyzed matrix, i.e., a urine matrix shows a range from 0.002 to 0.2 ng per mL [18,19], whilst the cell matrices display a higher LOQ up to 10 ng per mL [16]. This suggests that the matrix effect also depends on the specific matrix where the metabolites are contained. Comparing these studies, the cell matrices report a lower sensitivity compared to urine; this is important when applying this method in future experiments or assays. In fact, this observation spotlights the major limitation of this study: the quantitation has been performed semi-quantitively. Ion suppression in mass spectrometry negatively affects the analyte signal, and subsequently underestimates its quantitation, or it simply hampers its detection. Moreover, ion suppression may be limiting the detection of certain steroid compounds in several matrices, i.e., EV preparations. In consequence, this method should be utilized in matrices that facilitate the detection of the steroids. A matrix-spiked calibration is usually the appropriate method to quantify the absolute amounts of analytes in samples [30]. In this work, a calibration curve of the analyte standards was prepared in solution with 50% *v/v* methanol/water as a solvent. Such an approach cannot compute the absolute amounts of the analytes in tissue, since the matrix effect is not considered, however, a semi-quantitative approximation of the metabolites in tissues can be calculated. In this assay, the reported LOQ range lies between 0.50 and 50 nM (equivalent to 0.14 and 14.42 ng per mL) in solution, similar to previous studies. However, it is advised to use matrix-spiked curves in further experiments using this assay.

The time required to perform the chromatographic separation is typically long in the literature; they report runtimes from over 10 min up to 45 min [3,5,18,19,20,21,22,27]. Only the work of Quanson et al. [16] and Indapurkar et al. [17] described a methodology with a short runtime (4 to 5 min); however, they tested and applied the method solely in cell matrices: PCa and induced pluripotent stem cell lines, respectively. Indapurkar et al. [17] developed a methodology specific for estradiol-related metabolites and Quanson et al. [16] measured androgenic steroids using an ultra-performance convergence chromatography. In 2012, Maeda et al. accomplished the separation, detection and quantification of a panel of steroids in rat organs except in the liver, but using an HPLC system. For this reason, their sample preparation strategy demanded high volumes of extraction buffer—15 mL of acetonitrile per sample—and required a total run time of 11 min. In this work, the volumes are lower than 1 mL and the run time for different types of samples is lower than 10 min.

In order to test the performance of our methodology, we have measured steroid hormone analytes from several rat tissues: adrenal glands; testis; prostate; liver and brain. The data shown in Table 3 are in accordance with the fact that the pathway is tissue-dependent in regular physiological conditions. Two metabolites upstream of the pathway, pregnenolone and androstenedione, were quantified in the adrenal glands, but could not be quantified in prostate or brain. This hints that the adrenal glands are in charge of the conversion of cholesterol into the steroid compounds in complex organisms, such as rats; this is in line with previous findings in the literature [41,42,43]. Likewise, the adrenal glands are known to produce corticoid hormones. Our data confirms this, since corticosterone is quantified in a higher amount—three to four orders of magnitude—when compared to the prostate, brain and testicles. The adrenal glands also seem to accumulate androgens (Table 3); however, the presence of active androgens (DHT) is two-fold higher in the prostate compared to other tissues. Importantly, the ratio DHT/testosterone, which are the active and non-active paired androgens, was approximately 11 in prostate, while the adrenal gland and testis were below 1. Because the presence of the active androgen plays a physiological role in prostate, the ratio of DHT/testosterone was also higher in this tissue. 

Since the first urinary metabolomics attempts to analyze urinary samples and other biofluids, several methodologies have been developed during the last few years [18,19,20]. Nevertheless, none of the reported methodologies was optimal to assess the steroids in the EV sample preparations, tissues or body fluids in a fast and simple manner. Up to date, many of the studies have shown metabolomics in EVs [5,20,44], but none of them has reported the detection of steroid hormones in a targeted approach. A plausible explanation is that the identification and detection of compounds similar in molecular mass—even the same one in some cases—hampers the allocation of mass signals with the corresponding chromatographic peak. For those steroids, i.e., DHEA and testosterone, which share an empirical formula, the identification of each specific compound remains challenging using MS and the identification relies on chromatographic separation. 

Importantly, we have been able to quantify the steroid hormones in urine samples and derived uEV in a fast and simple manner. However, only one DHEAS was detected in the uEVS and cortisol, androstenedione and DHEAS were detected in the urine samples. These EVs were isolated by ultracentrifugation, including a washing step to avoid any contamination from the soluble fraction. The urine samples from a healthy man were collected on different days and different time of collection (morning and afternoon). The time collection was a parameter to be assessed from a metabolomics perspective, but we found out that inter-day variability also had a high impact on the analysis. Morning samples are considered to contain a higher concentration of steroid analytes coming from the prostate, possibly due to accumulation and leakage towards the urinary tract during the night. However, this trend was not described in our morning samples. The reason may be that urine sample U003 (Table S4, Supplementary Materials) was not available for metabolomics analysis; the analysis of the soluble fractions of urine (after uEV isolation), which includes U003, in the morning samples had a higher concentration of DHEAS. This highlights the importance of analyzing a larger cohort to obtain significant results non-dependent on a unique highly concentrated sample. 

In the end, this is a fast and sensitive method that was successfully applied for the detection and quantification of a panel of steroid hormone compounds in biological samples in 6 min runtime per sample. The sensitivity of this method makes it ideally suited for multiple in vivo applications. In this manuscript, we explored the analysis of steroids in several rat tissues and also in human urine and uEV samples. This has evident applications in profiling the metabolic status of patients suffering any hormone-dependent disease. It should be noted that the assay requires a longer cleanse step to wash the column out of the lipids and peptides when running a long experiment with many tissue samples. To our knowledge, this is the first hrLCMS-based method able to detect and quantify steroid hormones associated with EVs isolated from body fluids in a targeted approach.

## Figures and Tables

**Table 1:** Summary of the optimized method characteristics. The recoveries (±standard deviation) and matrix effect as signal loss (±standard deviation) of the extraction procedure in two different biological matrices (*n* = 6; biological matrix: DU145 cell) are reported. In addition, LOD and LOQ values of the analytes in the adequate fraction are compiled. LOD: Limit of detection; LOQ: Limit of quantification.

Analyte	Fraction	Recovery (%)	Matrix Effect (%)	LOD (nM)	LOQ (nM)
Pregnenolone	Organic	97.2 (±1.9)	25.2 (±3.1)	2.5 nM	10 nM
	Aqueous	-	24.0 (±2.8)		
DHEA	Organic	122.7 (±2.9)	37.7 (±5.7)	5.0 nM	50 nM
	Aqueous	-	28.0 (±6.2)	-	-
Androstenedione	Organic	102.2 (±3.2)	30.8 (±4.6)	0.25 nM	0.5 nM
	Aqueous	-	23.2 (±4.5)		
Estrone	Organic	103.7 (±3.8)	25.5 (±4.8)	5.0 nM	10 nM
	Aqueous	-	25.7 (±4.0)		
DHT	Organic	74.2 (±3.4)	23.1 (±3.9)	0.25 nM	1.0 nM
	Aqueous	-	23.4 (±2.9)		
Cortisol	Organic	114.3 (±3.8)	25.9 (±4.2)	0.5 nM	1.0 nM
	Aqueous	22.28 (±4.5)	17.6 (±4.7)		
Aldosterone	Organic	99.8 (±1.77)	18.7 (±4.3)	0.5 nM	2.5 nM
	Aqueous	-	17.7 (±5.1)		
Corticosterone	Organic	109.4 (±3.1)	25.1 (±3.6)	0.25 nM	1.0 nM
	Aqueous	-	20.2 (±3.2)		
Testosterone	Organic	126.9 (±1.7)	14.3 (±1.9)	0.25 nM	0.25 nM
	Aqueous	-	8.0 (±2.1)		
Pregnenolone sulfate	Organic	6.9 (±2.7)	25.2 (±3.1)	0.25 nM	1.0 nM
	Aqueous	94.8 (±1.9)	24.0 (±2.8)		
DHEAS	Organic	-	42.6 (±1.1)	0.25 nM	0.5 nM
	Aqueous	108.0 (±1.4)	42.5 (±0.1)		

**Table 2:** Summary of the recoveries using the optimized methodology in urine matrix. The recoveries (±standard deviation) of two different volumes (50 µL and 250 µL) of pre-pooled urine are reported (*n* = 3).

Analyte	Urine Volume	Recovery (%)
Pregnenolone	50 µL	92.4 (±3.6)
	250 µL	99.3 (±4.8)
Androstenedione	50 µL	93.0 (±3.9)
	250 µL	79.3 (±3.8)
Estrone	50 µL	94.2 (±3.3)
	250 µL	84.8 (±4.8)
DHT	50 µL	76.3 (±4.1)
	250 µL	71.2 (±3.76)
Cortisol	50 µL	87.0 (±3.0)
	250 µL	72.4 (±3.6)
Aldosterone	50 µL	110.7 (±2.9)
	250 µL	103.1 (±3.2)
Corticosterone	50 µL	96.2 (±2.8)
	250 µL	84.3 (±3.6)
Testosterone	50 µL	104.1 (±2.1)
	250 µL	96.3 (±5.1)
Pregnenolone sulfate	50 µL	54.9 (±1.5)
	250 µL	25.5 (±1.2)
DHEAS	50 µL	75.7 (±2.5)
	250 µL	44.0 (±4.2)

**Table 3:** Quantitation of three independent Wistar rat tissues: adrenal gland, prostate and brain. Adrenal glands of the same animal were titered independently, also, the prostate lobes of each rat. The averages in nmol per gram of tissue, standard deviations and coefficients of variation (%) of the three groups of samples are reported.

Analyte	Quantification (nmol/g Tissue)	Adrenal Gland	Prostate	Brain	Testicle	Serum (nM)
Pregnenolone	Amount	7.04	-	Detected	Detected	-
	St. dev.	3.74				
	%cv	53				
Androstenedione	Amount	5.97 × 10^−3^	-	Detected	1.45 × 10^−3^	Detected
	St. dev.	3.35 × 10^−3^			1.38 × 10^−3^	
	%cv	56			95	
DHT	Amount	3.47 × 10^−3^	7.57 × 10^−3^	Detected	2.70 × 10^−3^	Detected
	St. dev.	1.02 × 10^−3^	2.40 × 10^−3^		7.92 × 10^−4^	
	%cv	29	31		29	
Corticosterone	Amount	18.89	4.01 × 10^−3^	2.42 × 10^−2^	1.25 × 10^−3^	28.01
	St. dev.	10.05	5.15 × 10^−3^	7.04 × 10^−3^	7.98 × 10^−4^	3.31
	%cv	53	128	29	63	12
Cortisol	Amount	0.45	-	-	-	-
	St. dev.	0.19				
	%cv	43				
Testosterone	Amount	4.53 × 10^−3^	6.92 × 10^−4^	7.02 × 10^−4^	9.18 × 10^−3^	0.20
	St. dev.	1.47 × 10^−3^	2.36 × 10^−4^	4.29 × 10^−4^	4.53 × 10^−3^	0.02
	%cv	32	34	60	49	

**Table 4:** Quantitation of urine human samples (*n* = 6, U001 to U006, Table S4, Supplementary Materials). The isolated EV fraction are also included in the table. In the table, the three analytes detected in the urine-derived samples.

Sample	Collection Time	Androstenedione (nM)	Cortisol (nM)	DHEAS		
				Conc. (µM)	EV-Associated DHEAS (µM)	EV-Associated DHEAS in Urine (ppm)
Urine	Morning	2.25 (±0.92)	40.1 (±33.5)	0.36 (±0.16)	-	-
Urine	Afternoon	1.95 (±0.78)	35.7 (±0.7)	1.27 (±0.87)	-	-
SN100K	Morning	2.31 (±1.53)	29.9 (±14.7)	1.33 (±0.94)	-	-
SN100K	Afternoon	1.82 (±0.64)	31.4 (±0.7)	0.87 (±0.92)	-	-
P10K	Morning	-	-	-	1.75	0.90
P10K	Afternoon	-	-	-	0.76 (±0.08)	0.79 (±0.41)
P100K	Morning	-	-	-	6.17	3.19
P100K	Afternoon	-	-	-	0.74 (±0.01)	

Concentration (±standard deviation) of the analytes in urine and supernatant fraction of both morning and afternoon collected urine is shown. Absolute amount and relative amount (±standard deviation) of DHEAS is calculated in 50 mL of initial sample of both morning and afternoon collected urine.

## Data Availability

All data which support the reported results have been uploaded to figshare (https://figshare.com/articles/dataset/_/20231493) (accessed on 27 June 2022). The reference to this data was added as [32] in the text.

## Supplementary Materials

The following supporting information can be downloaded at: www.mdpi.com/article/10.3390/metabo12080714/s1, Supporting Information S1: Additional experimental details related to sample collection and characterization; Supporting Information S2: Table S1–S4 Supplementary tables with method optimization data and urine characterization; Supporting Information S3: Figure S1–S5 Supplementary figures including metabolomics network, method optimization results and urine characterization.
