# Supplementary material for: Simultaneous Quantification of Steroid Hormones Using hrLC-MS in Endocrine Tissues of Male Rats and Human Samples
Source: Metabolites. 2022 Jul 30;12(8):714. doi: 10.3390/metabo12080714 (PMC9414922; doi:10.3390/metabo12080714)
Supplement: Supplementary file 1 [file metabolites-12-00714-s001.zip › Supporting information S1.pdf]

Supporting information S1: Additional experimental details

Tissue and serum sample preparations from rats

Tissues and serum were obtained from wild-type (Wistar, RjHan:WI) rats (Janvier Labs, Le Genest-Saint-Isle, France). Rats were fed with sterile water and a standard diet ad libitum in a temperature- and light cycle-controlled animal facility following the Spanish Guide for the Care and Use of Laboratory Animals (RD 53/2013 - BOE-A-2013-1337). All rats underwent a liver perfusion procedure and were sacrificed by bleeding. Immediately after the perfusion, brain, prostate, testicles and adrenal gland tissues were removed and directly frozen at -80 ºC in dry ice for further use. Liver tissue was collected from animals in which liver perfusion could not be completed. Liver, prostate and testicle tissues were cut on dry ice and aliquoted into portions between 50 to 80 mg prior metabolite extraction. Adrenal glands were stored individually as each individual weighted approximately 80 mg. The exact weight of all tissue samples was calculated and utilised normalise the detected metabolites. Blood was collected during the perfusion procedure into a BD Microtainer® blood collection tube with separator gel (BD, Franklin Lakes, NJ). Then, it was centrifuged at 8,000g for 15 min and the serum fraction transferred into a clean Eppendorf® tube prior storage at -80 ºC for further processing.

Human urine samples

All urine samples were obtained from a healthy male on either the morning (first urine of the day after fasting) or the afternoon (after lunch, approximately at 4pm) to study circadian variations. Each collection time has three biological replicates collected in independent days by spontaneous micturition. Approximately 80 mL of urine were collected of which 50 mL were centrifuged at 2,000g for 10 min, filter sterilised (0.22 µm pore size) and immediately frozen at -80 ºC for further processing. An aliquot of the original urine was also stored at -80ºC for further analysis. The six different urine samples were characterized regarding the following parameters: blood and ketone bodies presence in urine, glucose concentration, pH value and density (Sup. Table 3). Dip-and-read stripes were introduced into thawed urines samples and physicochemical parameters were measured with an OneStepTM Plus Stripe Urine Analyser (Henry Schein Inc., Melville, NY).

EV isolation procedure

Urine samples were thawed at room temperature and centrifuged for 5 min at 2,000g to remove any precipitate . Then, they were centrifuged at 10,000 g for 30 min to obtain a pellet (P10K fraction) containing EVs of bigger size than in the remaining supernatant. In a next step, the urine supernatant was ultracentrifuged at 100,000g for 90 min; the resulting pellet (Fraction P100K) contains EVs of smaller size than P10K. Fraction P10K and P100K were both washed in 50 mL of phosphate-saline buffer (PBS) and ultracentrifugated at 100,000g for 90 min. Afterwards, both fractions were resuspended in 50 µL of PBS and stored at -80 ºC together with the urine supernatant (SN100K) of P100K for further analysis.

Western blot Analysis

An aliquot of 6 µl of each urinary EV (uEV) preparation was loaded and separated under non-reducing conditions in 4–12% Bis-Tris Protein gels (Invitrogen Inc., Waltham, MA). Western blotting was performed to determine the presence and relative amount of uEVs in each sample and fraction, for this reason only the approximately 10% of each uEV isolated fraction was utilized. In brief, the proteins were transferred to nitrocellulose membranes and then, they were blocked for 1 h (in 5% non-fat milk and 0.1% Tween-20 PBS solution). Then, the primary antibody was incubated overnight (approximately 16 h) at 4 ºC, washed and incubated for 1 h with a secondary HRP-conjugated antibody at room temperature. The primary antibodies used in this study were: MoαCD63 (clone H5C6) purchased from Developmental Studies Hybridoma Bank (Iowa, IA), MoαCD9 (clone 209306) from R&D Systems (Minneapolis, MN), RbαAQP2 (clone A7310) obtained from Sigma-Aldrich (St. Louis, MO), RbαCOX-IV (clone 3E11) from Cell Signaling Technology (Danvers, MA), MoαCD10 (clone F-4) from Santa Cruz Biotechnology Inc. (Dallas, TX), and finally, RbαAnnexin V (ab14196) and RbαLAMP2A (clone EPR4207(2)) were both purchased from Abcam (Cambridge, UK). Jackson ImmunoResearch, Inc. provided the mouse and rabbit HRP-conjugated secondary antibodies. A Clarity Western ECL kit from Bio-Rad (Hercules, CA) was utilized for the chemiluminescence detection of bands. Either by scanning AmershamTM HyperfilmTM MP photosensible films (Cytiva, Uppsala, Sweden) or using a luminescent image analyser ImageQuantTM LAS 4000 (GE Healthcare, Chicago, IL), the proteins in the nitrocellulose membranes coming from different urine samples and fractions were identified.
