# Supplementary material for: Simultaneous Quantification of Steroid Hormones Using hrLC-MS in Endocrine Tissues of Male Rats and Human Samples
Source: Metabolites. 2022 Jul 30;12(8):714. doi: 10.3390/metabo12080714 (PMC9414922; doi:10.3390/metabo12080714)
Supplement: Supplementary file 1 [file metabolites-12-00714-s001.zip › Supporting information S2.pdf]

Supporting information S2: Supplementary tables

Table S1. Summary of the six gradient utilised in optimization rounds to establish the best methodology with respect to peak intensity and analyte separation.

	**Gradient 1**		**Gradient 2**		**Gradient 3**		**Gradient 4**		**Gradient 5**		**Gradient 6**	
**Time (min)**	% A	% B	% A	% B	% A	% B	% A	% B	% A	% B	% A	% B
**0**	95	5	90	10	85	15	80	20	80	20	70	30
**3.8**	-	-	10	90	10	90	10	90	20	80	20	80
**4**	1	99	1	99	1	99	1	99	1	99	1	99
**4.8**	1	99	1	99	1	99	1	99	1	99	1	99
**5**	95	5	90	10	85	15	80	20	80	20	70	30

Table S2. Summary z-spray source parameters optimized for *m/z* 556.2771 in ES+ and ES-.

Parameter	ES+	ES-
Capillary voltage	1.00 kV	2.00 kV
Sampling cone voltage	25	40
Source Offset	80	80
Source temperature	120 ºC	120 ºC
Desolvation temperature	450 ºC	450 ºC
Desolvation gas flow	5.00 L/h	5.00 L/h
Cone gas flow	1,000 L/h	600 L/h
Nebuliser	6.00 bar	6.00 bar

Table S3. Summary of the five different extraction buffers tested in the recovery efficiency experiments.

Extraction buffer	% methanol	% H2O	pH modifier
Buffer 1	50	50	None
Buffer 2	50	49	1% formic acid
Buffer 3	25	75	None
Buffer 4	25	74	1% formic acid
Buffer 5	50	49.975	1 mM ammonia

The buffers differ in their methanol content and the compound utilised to tune the pH of the buffer (pH modifier).

Table S4. Summary of the six urine samples collected to test the metabolomics assay.

**Parameter**	**U001**	**U002**	**U003**	**U004**	**U005**	**U006**
**Date (yyyymmdd)**	20210521	20210514	20210423	20210524	20210428	20210505
**Collection time**	Afternoon	Morning	Morning	Afternoon	Morning	Afternoon
**Blood**	NEG	NEG	NEG	NEG	NEG	NEG
**Ketone bodies**	NEG	NEG	NEG	NEG	NEG	NEG
**Glucose**	NEG	NEG	NEG	NEG	NEG	NEG
**pH**	6	5	6	6	6	5
**Density (g/mL)**	1.025	1.025	1.020	1.015	1.025	1.025

Collection time and urine characteristics of each sample are reported.
