# Supplementary material for: Simultaneous Quantification of Steroid Hormones Using hrLC-MS in Endocrine Tissues of Male Rats and Human Samples
Source: Metabolites. 2022 Jul 30;12(8):714. doi: 10.3390/metabo12080714 (PMC9414922; doi:10.3390/metabo12080714)
Supplement: Supplementary file 1 [file metabolites-12-00714-s001.zip › Supporting_information S3.pdf]

Supporting information S3: Supplementary Figures

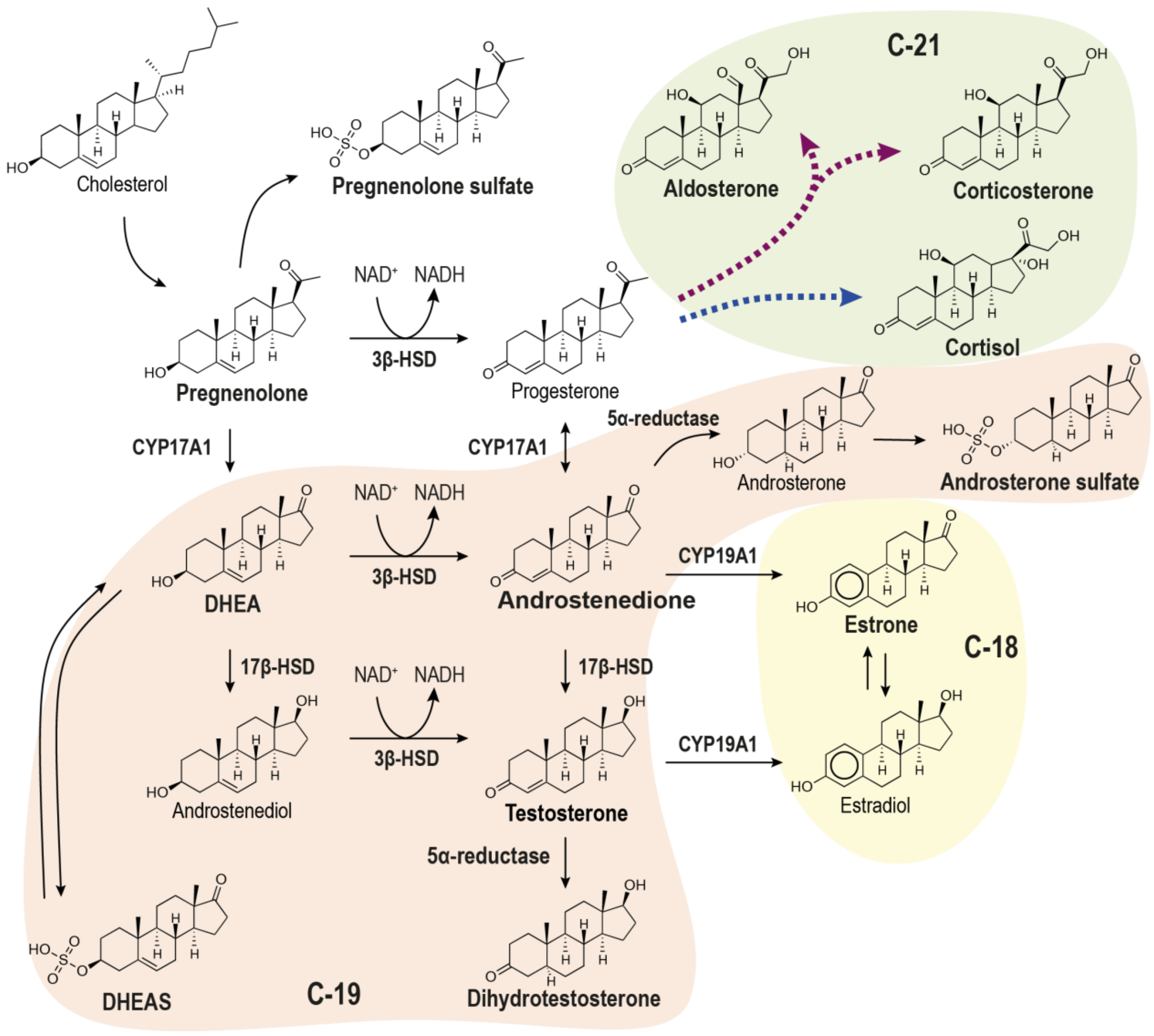

Figure S1. Schematic representation of the steroid hormones biosynthesis pathway[7,8]. In bold, relevant metabolites of the metabolic network (chosen as analytical standards) are highlighted. The dotted lines indicate a pathway diversion towards C-21 steroid compounds; mineralocortoids and glycorticoids which are depicted in purple and blue, respectively. Each arrow represents a reaction driven by an enzyme or groups of enzymes while a dotted line indicates a metabolic path but no direct reactions. Modules of steroid hormones are categorised in three groups: C-18 in yellow, C-19 in orange and C-21 in green. DHEA, Dehydroepindrosterone; DHEAS, Dehydroepiandrosterone sulphate.

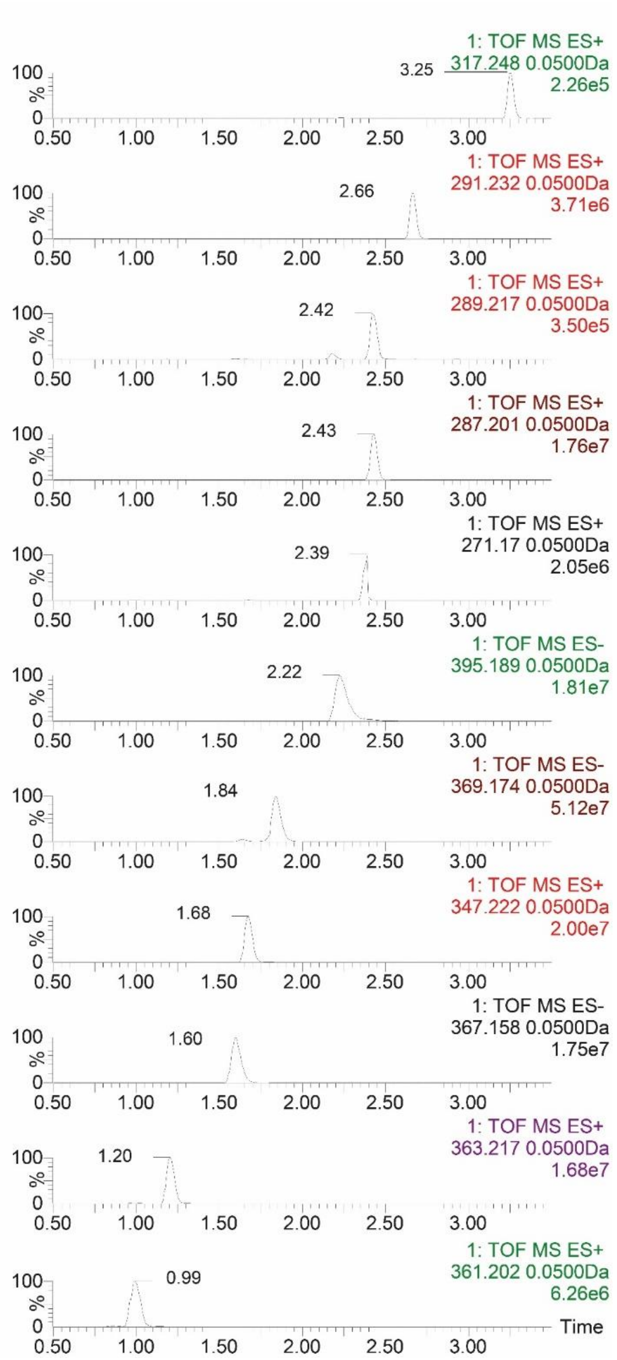

Figure S2. Extracted ion current chromatograms of the analytes from a mixture at 10 uM concentration of each standard. In each chromatogram, the optimal ES (+ or -), m/z value and signal intensity of detection are indicated. They are ordered by decreasing retention time. From top to bottom pregnenolone, dihydrotestosterone, DHEA, androstenedione, estrone, pregnenolone sulphate, androsterone sulphate, corticosterone, DHEAS, cortisol and aldosterone. In green, the parameters of the analytes detected in ES- mode; in dark orange, the parameters of the analytes detected in ES+ mode.

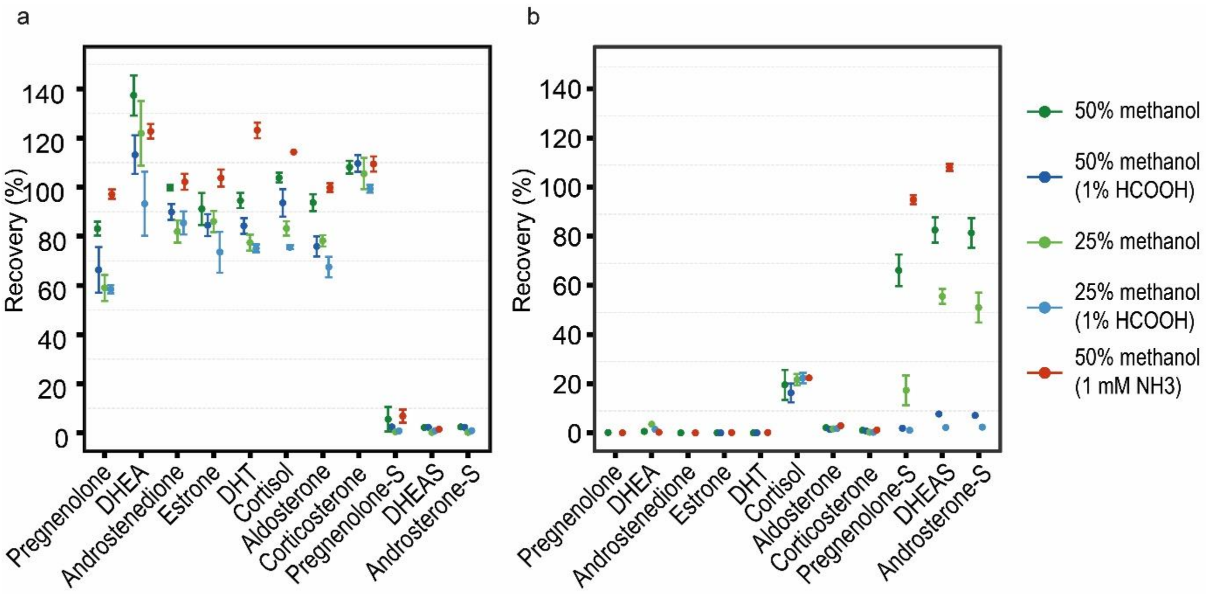

Figure S3. Recoveries (± standard deviation) of the selected panel of standard analytes are shown (n=6). For each analyte, the recoveries using different extraction buffers are depicted. In green, the results of an extraction using only a mixture with the solvent (50% methanol dark green and 25% methanol light green). In blue, the results with extraction buffers containing 1% of formic acid (50% methanol dark blue and 25% methanol light blue). Recoveries obtained with a 50% methanol (1mM NH_3) extraction are depicted in red. a, analyte recoveries of the organic fraction. b, analyte recoveries of the aqueous fraction.

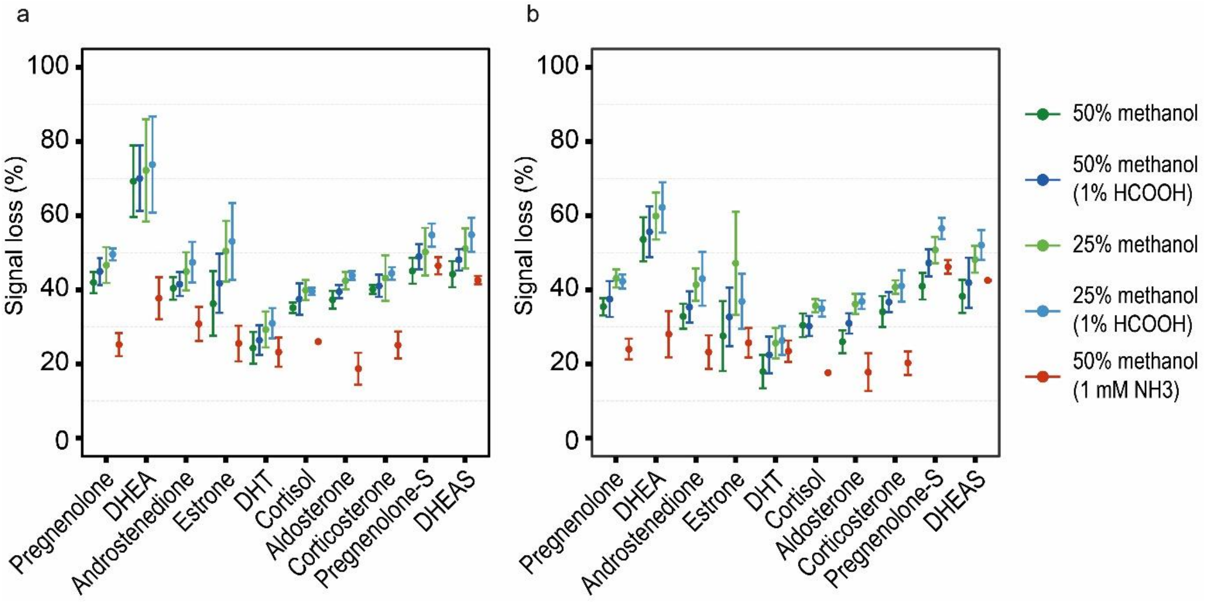

Figure S4. Matrix effect in the detection and quantification of the panel of analytes represented as signal loss (± standard deviation). The matrix effect per analyte in both organic (a) and aqueous fraction (b) is depicted (n=6). In green, the signal loss after extracting metabolites with mixture of only a solvent (50% methanol dark green and 25% methanol light green). In blue, the results with extraction buffers containing 1% of formic acid (50% methanol dark blue and 25% methanol light blue). Matrix effect after a 50% methanol (1mM NH_3) extraction is depicted in red.

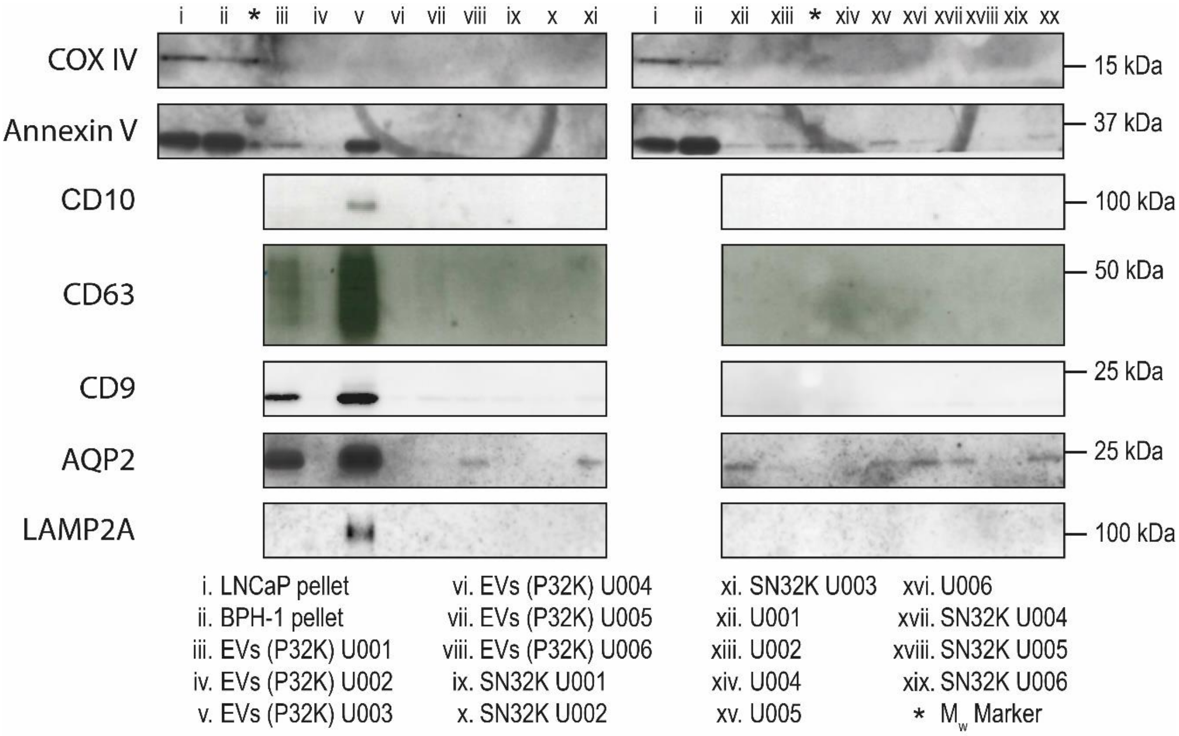

Figure S5. Western blot of the urine-derived fractions of the Sup. Table 4 samples. Each lane is numbered and each number is related to a specific fraction. COX IV, Annexin V, CD10, CD63, CD9, AQP2 and LAMP2 antibodies were tested for all the fractions. On the right, the molecular weight (in kDa) of each specific antibody result. Asterisc (*): protein ladder (Marker).
